# HER2 genomic amplification in circulating tumor DNA from patients with cetuximab-resistant colorectal cancer

**DOI:** 10.18632/oncotarget.6498

**Published:** 2015-12-02

**Authors:** Naoki Takegawa, Kimio Yonesaka, Kazuko Sakai, Hiroto Ueda, Satomi Watanabe, Yoshikane Nonagase, Tatsuya Okuno, Masayuki Takeda, Osamu Maenishi, Junji Tsurutani, Taroh Satoh, Isamu Okamoto, Kazuto Nishio, Takao Tamura, Kazuhiko Nakagawa

**Affiliations:** ^1^ Department of Medical Oncology, Kinki University School of Medicine, Osaka, Japan; ^2^ Department of Genome Biology, Kinki University School of Medicine, Osaka, Japan; ^3^ Department of Pathology, Kinki University School of Medicine, Osaka, Japan; ^4^ Department of Frontier Science for Cancer and Chemotherapy, Osaka University Graduate School of Medicine, Osaka, Japan; ^5^ Center for Clinical and Translational Research, Kyushu University, Kyushu, Japan

**Keywords:** colorectal cancer, cetuximab, acquired resistance, human epidermal growth factor receptor 2, ctDNA

## Abstract

**Background:**

Patients with metastatic colorectal cancer (mCRC) harboring wild-type KRAS benefit from epidermal growth factor receptor (EGFR)-targeted therapy. However, patients who are treated with anti-EGFR antibodies will eventually develop the resistance to those agents. HER2 amplification is one of the mechanisms conferring resistance to anti-EGFR antibody therapy and could therefore be a potential therapeutic target. The aim of this study was to detect HER2 amplification in circulating tumor DNA (ctDNA) from patients with CRC and acquired resistance to anti-EGFR antibody therapy.

**Results:**

Our data showed that 22% (4/18) of patients in the cohort exhibited HER2 amplification. One of these patients was found to be positive for HER2 amplification in matched tumor specimens collected after cetuximab therapy, at which point the patient had acquired cetuximab resistance, despite being negative for HER2 amplification prior to therapy.

**Methods:**

We analyzed plasma ctDNA using digital polymerase chain reaction (PCR) from 18 patients with CRC, who had been treated with anti-EGFR antibody-based therapy (cetuximab) and subsequently acquired resistant cetuximab. HER2 gene copy number was analyzed using fluorescence in situ hybridization in tumor samples before and after acquisition of resistance to cetuximab-based therapy.

**Conclusion:**

Analysis of plasma ctDNA by digital PCR could be useful for detecting HER2 amplification in patients with CRC who were resistant to anti-EGFR antibody therapy.

## INTRODUCTION

Colorectal cancer (CRC) is the third most common cancer in the world. Advances in systemic chemotherapies have led to significant improvements in survival for patients with metastatic CRC [1, 2]. Indeed, recent research efforts have focused on the development of agents targeting the epidermal growth factor receptor (EGFR), which is frequently overexpressed in colorectal cancer and contributes cancer cell proliferation, metastasis, and angiogenesis [3]. Anti-EGFR antibody therapies, including cetuximab and panitumumab, have improved the prognosis of patients with CRC, particularly in patients with the wild-type *KRAS* gene, in which these agents exhibit enhanced efficacy [4–7]. KRAS functions downstream of EGFR, and its spontaneous activation due to mutation promotes cell proliferation despite the presence of anti-EGFR antibody [8].

However, the clinical efficacy of anti-EGFR antibody therapy is eventually limited by the development of acquired resistance. Several mechanisms for acquired resistance to anti-EGFR antibody therapy have been identified in CRC. For example, *KRAS* and *NRAS* genomic alternations may evolve under anti-EGFR antibody therapy, resulting in resistance to these therapies [9][10]. Alternatively, EGFR ectodomain mutations, such as S492R, have been shown to prevent anti-EGFR antibodies, particularly cetuximab, from binding with EGFR, thereby conferring resistance to this therapy [11]. Furthermore, our previous studies have shown that HER2 genomic amplification causes resistance to cetuximab in a preclinical model and in clinical samples [12]. Specifically, HER2 amplification was shown to evolve in non-small cell lung cancer (NSCLC) and CRC cell lines after prolonged exposure to cetuximab. Moreover, HER2 signaling bypasses cell proliferation signals derived from EGFR under EGFR inhibition with cetuximab. Notably, HER2 genomic amplification was shown to evolve in CRC tumors also after acquisition of resistance to cetuximab, despite the absence of HER2 amplification prior to cetuximab therapy. This resistance could be overcome using HER2 inhibitors, such as trastuzumab and lapatinib.

Repeated sampling of tumors is helpful to determine how tumors develop resistance after systemic therapy. However, this approach has limitations because of the invasiveness of biopsy procedures and tissue heterogeneity. Circulating tumor DNA (ctDNA) originating from tumor cells may reflect the pathological condition of the original tumor [13]. ctDNA can be obtained less invasively than tumor biopsies and can provide information regarding systematic tumor characteristics. Therefore, ctDNA may be useful for diagnosing how cancer cells acquire resistance. For example, a previous study detected the development of KRAS mutations in ctDNA from some patients with CRC who had been treated with anti-EGFR antibody therapy [14]. Therefore, it is possible that HER2 amplification may be detected in ctDNA from patients with CRC who have developed resistance to anti-EGFR antibody therapy. HER2 genomic amplification is rare in CRC [15], but is more frequent in patients with breast cancer [16] and can be detected in ctDNA [17].

In this study, we aimed to detect HER2 amplification in ctDNA from patients with CRC who acquired resistance to anti-EGFR antibody therapy.

## RESULTS

### Patient characteristics

Plasma samples were obtained from 18 patients with histologically confirmed metastatic CRC who were being treated with cetuximab-based therapy. The patients' baseline characteristics, including age, sex, primary tumor site, drug regimen, best overall response, and progression-free survival (PFS), are summarized in Table 1. All patients had tumors with wild-type KRAS; had been treated with fluoropyrimidine, oxaliplatin, irinotecan, and bevacizumab; and were refractory to those agents prior to cetuximab-based therapy. Eight patients achieved partial response, and 10 patients had durable tumor stabilization for more than 10 weeks following initiation of cetuximab-based therapy. All patients continued cetuximab-based therapy until tumor progression (maximum duration: 784 days). Median PFS was 182.5 days, and four patients had no tumor progression for more than 1 year.

**Table 1:** Patients Characteristics

Age	51–80
Sex	
Male	13
Female	5
Primary Site	
Rectum	8
Sigmoid	7
Decending	2
Transverse	1
Ascending, Cecum	0
Regimen	
Cetuximab+Irinotecan	11
Cetuximab alone	6
Cetuximab+FOLFIRI	1
Best overall response	
CR	0
PR	8
SD	10
PD	0
PFS(days)	70-554 (182.5)

Abbreviations: CR, complete response; PR, partial response; SD, stable disease; PD, progressive disease; PFS, progression-free survival

### Relative HER2 copy number ratio in ctDNA

Relative HER2 copy number ratios varied among all samples and are summarized in Table 2 (*n* = 18; median: 1.09; range: 0.94–5.18). The cut-off value for the relative HER2 copy number ratio in plasma samples was set at 1.25, as described previously [17]. Samples from four patients (22%, patient numbers 5, 10, 14, and 17) were classified as HER2-positive cases. Among these four HER2-positive patients, three achieved partial response and had durable PFS longer than 5 months before plasma was obtained. Additionally, we evaluated the extracellular domain of HER2 in serum using chemiluminescent immunoassays [12]. The expression level of the extracellular domain of HER2 varied among these patients (*n* = 16; median: 11.4 ng/mL; range: 5.2–17.9 ng/mL). One patient (#10) with *HER2* copy number amplification had the highest concentration of the extracellular domain of HER2 among all patients. However, there was no significant correlation between relative *HER2* copy number ratios in ctDNA and the concentration of the extracellular domain of HER2.

**Table 2:** HER2 ratio with digital PCR and HER2 ECD in plasma

No.	Relative quantitation of HER2 gene	HER2 ECD protein, ng/mL
1	1.23	11.4
2	0.97	10.1
3	0.94	12.6
4	1.08	8.7
5	5.18*	13..5
6	1.07	15.9
7	1.20	9.0
8	1.09	5.2
9	1.09	15.8
10	1.33*	17.9
11	1.09	12.2
12	1.08	9.9
13	1.09	7.7
14	1.29*	13.1
15	1.11	-
16	1.01	15.3
17	1.40*	9
18	1.15	-

* greater than cutoff value, 1.25

Abbreviations: HER2, human epidermal growth factor receptor 2; PCR, polymerase chain reaction; ECD, extra cellular domain

### HER2 genomic amplification in tumors after acquisition of resistance to cetuximab-based therapy

**Figure 1:**
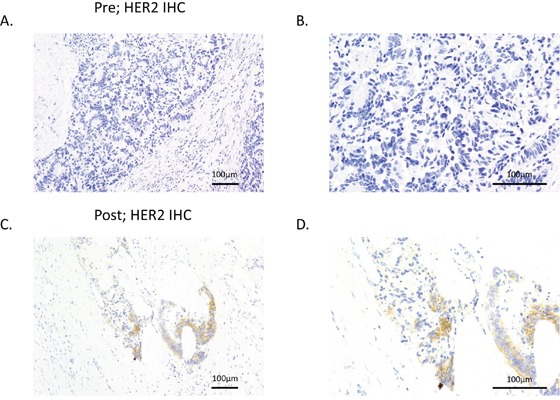
HER2 expression was increased after resistance to cetuximab was acquired HER2 expression levels were evaluated in tumor samples from patient #5 prior cetuximab therapy **A, B.** and after acquisition of resistance to cetuximab therapy **C, D.** using IHC. A and C show low-magnification images, and B and D show high-magnification images.

**Figure 2:**
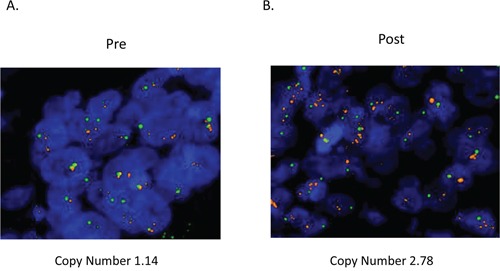
HER2 genomic amplification was observed in tumors after acquisition of resistance to cetuximab HER2 copy numbers were evaluated by FISH in tumors before and after acquisition of resistance to cetuximab in patient #5. Red: HER2; green: CEP17.

To examine the concordance of HER2 amplification between plasma ctDNA and tissue samples, we rebiopsied the metastatic lesion in patient #5 after acquisition of resistance to cetuximab therapy. HER2 expression levels were evaluated with immunohistochemistry (IHC), and *HER2* genomic copy numbers were measured by fluorescence in situ hybridization (FISH) in tumors. IHC showed evidence of HER2 overexpression, scoring 2+ according to the diagnostic criteria for colorectal cancer [18], this feature was detected in the tumor obtained after acquisition of cetuximab resistance. In contrast, HER2 was not expressed in the primary lesion prior to cetuximab therapy (Figure 1). According to the diagnostic criteria for colorectal cancer, FISH analysis also revealed that *HER2* amplification was acquired along with cetuximab resistance after cetuximab therapy (*HER2* copy numbers: before therapy, 1.14 and after therapy, 2.78; Figure 2).

## DISCUSSION

In the current study, we detected *HER2* amplification in plasma ctDNA from patients with cetuximab-resistant CRC. Moreover, we found that one patient with plasma *HER2* copy number elevation exhibited *HER2* amplification in the tumor after acquisition of resistance to cetuximab. These results suggested that ctDNA could potentially provide information regarding the effects of *HER2* amplification on resistance to cetuximab in patients with CRC.

Several types of acquired resistance mechanisms for anti-EGFR antibody therapy have been described [19], and some related genomic alterations can be detected in ctDNA [9][14]. Consistent with our current study, Siravegna et al reported that *HER2* amplification could be detected in plasma ctDNA from patients with CRC who were resistant to anti-EGFR antibody therapy. Importantly, they also identified genomic alterations, including *KRAS, NRAS, MET, FLT3, EGFR,* and *MAP2K1,* in plasma ctDNA from those patients [20]. Taken together, these data suggest ctDNA may provide an exhaustive genomic explanation, including *HER2* amplification, for anti-EGFR antibody resistance in CRC.

Previous studies have reported the *HER2* amplification frequency ranges from 3% to 5.8% at the time of CRC diagnosis [15][21]. However, in the current study, four patients (22%) were classified as positive for plasma *HER2* DNA. This discrepancy regarding the frequency of *HER2* amplification may be explained by the limited samples size in this study or the clonal evolution of *HER2* after anti-EGFR antibody treatment. Other studies have reported the emergence of mutant *KRAS* alleles or *MET* amplification following anti-EGFR antibody therapy in patients with CRC [9][22]. However, in the current study, we could not evaluate *HER2* amplification in plasma ctDNA in a time-dependent manner. Moreover, for patient #5, tumor samples did not exhibit *HER2* amplification at the time of diagnosis, despite the appearance of *HER2* amplification in both the tumor and plasma after acquisition of resistance to cetuximab. In the current study, some patients with HER2 amplification in ctDNA could not undergo rebiopsy after acquisition of cetuximab resistance. Generally, patients with advanced colorectal cancer refuse to undergo additional biopsies because of the invasiveness of the method and the lack of substantial benefits. In contrast to rebiopsy, analysis of ctDNA could facilitate decisions regarding optimal treatment strategies in a less invasive manner.

There were no significant correlations between relative HER2 copy number ratios in ctDNA and the concentration of the extracellular domain of HER2. In particular, patient #5 exhibited HER2 amplification in plasma and in the tumor after cetuximab treatment, despite the reduced HER2 ECD level. The extracellular domain of HER2 is shed by protease-like ADAM10 in cancer cells. Alternatively, HER2 ECD could be metabolized systemically. These additional factors may affect HER2 ECD levels; therefore, HER2 ECD levels are not correlated with HER2 amplification in ctCDA.

Patients with *HER2* amplification were resistant to anti-EGFR antibody therapy, but may benefit from other treatment strategies. Preclinical studies have shown that *HER2*-targeting therapy, including lapatinib or trastuzumab, can overcome cetuximab resistance in NSCLC and CRC cells with HER2 amplification [12]. Furthermore, Siena et al reported that trastuzumab and lapatinib combination therapy induced a partial response in seven of 22 patients (32%) with HER2-positive CRC who were resistant to anti-EGFR antibody therapy [24]. Additionally, Deeken et al reported that the combination of cetuximab and lapatinib provided a partial response in some patients with CRC who were resistant to anti-EGFR antibody therapy [25]. Therefore, these data suggest that evaluation of HER2 in ctDNA may be helpful for determining the optimal therapy involving HER2-targeting combination therapy rather than anti-EGFR antibodies alone in patients with CRC.

## MATERIALS AND METHODS

### Study patients

Plasma samples were obtained from 18 patients with histologically confirmed metastatic CRC who were treated with cetuximab-based therapy at Kinki University Hospital between November 2008 and October 2011. RESIST version 1.1 was used to assess best overall response. Written, informed consent was obtained from all participants. The study protocols were approved by the ethics committee of Kinki University Hospital.

### FISH analyses

Cell suspensions were dropped onto precleaned slides and air-dried. Three-day-old slides were analyzed using the dual-color FISH assay with the PathVysion DNA probe set. The slides were incubated in 70% acetic acid for 40 s, digested in 0.008% pepsin/0.01 M HC1 at 37°C for 5 min, fixed in 1% formaldehyde for 10 min, and dehydrated in an ethanol series. Formalin-fixed, paraffin embedded (FFPE) tissue sections from patients with CRC were subjected to dual-color FISH using the PathVysion probe (LSI HER2 SO/CEP17 SG, Abbott Molecular). Initially, the slides were incubated from 2 h to overnight at 56°C, deparaffinized in Citri-Solv, and washed in 100% ethanol for 10 min. The slides were sequentially incubated in 2× SSC at 75°C for 10–24 min, digested in 0.25 mg/m: proteinase K/2× SSC at 45°C for 10–24 min, washed in 2× SSC for 5 min, and dehydrated in ethanol. The probe was applied according to the manufacturer's instructions to the selected hybridization area, which was covered with a glass coverslip and sealed with rubber cement. DNA denaturation was performed for 15 min at 85°C, and hybridization was allowed to occur at 37°C for 12–24 h. Post-hybridization washes were performed sequentially with 2× SSC/0.3% NP40 (pH 7.0–7.5) at 73°C for 2 min and 2× SSC for 2 min, and samples were dehydrated in ethanol. Chromatin was counterstained with DAPI (0.3 μg/mL) in Vectashield mounting medium (Vector Laboratories). Analysis was performed on an epifluorescence microscope using single interference filters sets for green (FITC), red (Texas red), blue (DAPI), dual (red/green), and triple (blue, red, and green) band-pass filters.

### IHC assessment of HER2 expression

Sections of archived formalin-fixed, paraffin-embedded tissue (4 μm thick) were placed on slides coated with polylysine. After deparaffinization and blocking of endogenous peroxidase, HER2 immunostaining was performed using rabbit anti-human c-erbB-2 as a primary antibody (Dako Corp, Carpinteria, CA, USA) at a 1:100 dilution. Primary antibody binding was assessed using the Dako Quick-Staining, Labeled Streptavidin-Biotin System (Dako) and followed by the addition of diaminobenzidine as a chromogen.

### Assessment of HER2 extracellular domain concentrations

Plasma was obtained from 16 patients with CRC after the acquisition of resistance to cetuximab. The HER2 extracellular domain was measured with a chemiluminescent immunoassay according to the manufacturer's recommendations (Siemens Healthcare Diagnostics).

### ctDNA extraction

Plasma ctDNA was purified using a QIAamp Circulating Nucleic Acid Kit (Qiagen, Valencia, CA). HER2 copy number was measured using the QX100 Droplet Digital PCR System in accordance with the manufacturer's instructions (Bio-Rad, Hercules, CA, USA). The relative *HER2* copy number ratio was calculated relative to the control *EFTUD2* gene, as previously described [17]. For *HER2* copy number assays, the primer sequences were as follows: *HER2* forward, 5′-ACAACCAAGTGAGGCAGGTC-3′; *HER2* reverse, 5′-GTATTGTTCAGCGGGTCTCC-3′; *HER2* probe, 5′-/56-FAM/AGGCACCCA/ZEN/GCTCTTTGAGG ACAAC/3IABkFQ/-3′; *EFTUD2* forward, 5′-GGTCTTGCCAGACACCAAAG-3′; *EFTUD2* reverse, 5′-TGAGAGGACACACGCAAAAC-3′; *EFTUD2* probe, 5′-/5HEX/TCCAGGTAG/ZEN/GACATCCTTTGGCTTT/3IAB kFQ/-3′. PCR was performed using the following cycling conditions: 95°C for 10 min, 40 cycles of 94°C for 30 s, and 58°C for 90 s, followed by enzyme deactivation at 98°C for 10 min. After thermal cycling, the plates were transferred to a Droplet reader. We aimed to obtain at least 100 droplets for *HER2* and *EFTUD2* to accurately assess the ratio. Digital PCR data were analyzed using the QuantaSoft analytical software package (Bio-Rad). The copy number of each gene (*HER2* and *EFTUD2*) was estimated from the Poisson distribution. *HER2* amplification with digital PCR was defined as a *HER2* ratio (*HER2/EFTUD2* copy number ratio) of 1.25 [17].

## SUPPLEMENTARY FIGURES AND TABLES


