# Supplementary material for: HER2 genomic amplification in circulating tumor DNA from patients with cetuximab-resistant colorectal cancer
Source: Oncotarget. 2015 Dec 2;7(3):3453–60. doi: 10.18632/oncotarget.6498 (PMC4823119; doi:10.18632/oncotarget.6498)
Supplement: Supplementary file 1 [file oncotarget-07-3453-s001.pdf]

SUPPLEMENTARY TABLE

Supplementary Table S1:

ID	Age	Sex	Primary site	Rejimen	Best Objective Response	Duration of treatment	PFS
1	60	M	Descending	Cetuximab+Irinotecan	PR	784	416
2	78	M	Rectum	Cetuximab alone	PR	278	182
3	55	M	Rectum	Cetuximab+Irinotecan	SD	96	97
4	58	M	Rectum	Cetuximab+Irinotecan	PR	181	183
5	75	F	Sigmoid	Cetuximab alone	PR	265	147
6	64	M	Descending	Cetuximab+Irinotecan	SD	384	231
7	80	M	Sigmoid	Cetuximab+Irinotecan	SD	70	70
8	51	F	Sigmoid	Cetuximab alone	SD	104	106
9	62	M	Rectum	Cetuximab+Irinotecan	SD	433	434
10	64	F	Sigmoid	Cetuximab+Irinotecan	PR	216	215
11	70	M	Rectum	Cetuximab+Irinotecan	SD	114	114
12	72	M	Transverse	Cetuximab alone	SD	174	180
13	54	F	Sigmoid	Cetuximab+Irinotecan	SD	125	124
14	74	M	Rectum	Cetuximab+Irinotecan	PR	447	446
15	56	M	Rectum	Cetuximab+Irinotecan	PR	580	554
16	60	M	Rectum	Cetuximab alone	PR	300	300
17	57	F	Sigmoid	Cetuximab alone	SD	287	286
18	57	M	Sigmoid	Cetuximab+FOLFIRI	SD	76	74
